# Narrow-Leafed Lupin (*Lupinus angustifolius*) β1- and β6-Conglutin Proteins Exhibit Antifungal Activity, Protecting Plants against Necrotrophic Pathogen Induced Damage from *Sclerotinia sclerotiorum* and *Phytophthora nicotianae*

**DOI:** 10.3389/fpls.2016.01856

**Published:** 2016-12-09

**Authors:** Jose C. Jimenez-Lopez, Su Melser, Kathleen DeBoer, Louise F. Thatcher, Lars G. Kamphuis, Rhonda C. Foley, Karam B. Singh

**Affiliations:** ^1^The Institute of Agriculture, The University of Western Australia, PerthWA, Australia; ^2^Department of Biochemistry, Cell and Molecular Biology of Plants, Estacion Experimental del Zaidin, Spanish National Research CouncilGranada, Spain; ^3^Centre for Environment and Life Sciences, Agriculture and Food, Commonwealth Scientific and Industrial Research Organisation, FloreatWA, Australia

**Keywords:** 7S globulins, fungal pathogen, legume, oxidative stress, plant defense, seed storage protein, vicilins

## Abstract

Vicilins (7S globulins) are seed storage proteins and constitute the main protein family in legume seeds, particularly in narrow-leafed lupin (*Lupinus angustifolius* L.; NLL), where seven vicilin genes, called β1- to β7-conglutin have been identified. Vicilins are involved in germination processes supplying amino acids for seedling growth and plant development, as well as in some cases roles in plant defense and protection against pathogens. The roles of NLL β-conglutins in plant defense are unknown. Here the potential role of five NLL β-conglutin family members in protection against necrotrophic fungal pathogens was investigated and it was demonstrated that recombinant purified 6xHis-tagged β1- and β6-conglutin proteins exhibited the strongest *in vitro* growth inhibitory activity against a range of necrotrophic fungal pathogens compared to β2, β3, and β4 conglutins. To examine activity *in vivo*, two representative necrotrophic pathogens, the fungus *Sclerotinia sclerotiorum* and oomycete *Phytophthora nicotianae* were used. Transient expression of β1- and β6-conglutin proteins in *Nicotiana benthamiana* leaves demonstrated *in vivo* growth suppression of both of these pathogens, resulting in low percentages of hyphal growth and elongation in comparison to control treated leaves. Cellular studies using β1- and β6-GFP fusion proteins showed these conglutins localized to the cell surface including plasmodesmata. Analysis of cellular death following *S. sclerotiorum* or *P. nicotianae* revealed both β1- and β6-conglutins suppressed pathogen induced cell death *in planta* and prevented pathogen induced suppression of the plant oxidative burst as determined by protein oxidation in infected compared to mock-inoculated leaves.

## Introduction

Plants are under constant exposure to potential microbial pathogens. One of the mechanisms they employ to defend themselves is via the production of bioactive antimicrobial proteins (AMPs). In addition to plants, other organisms may produce a diverse array of AMPs for defense purposes and these can confer a high level of antimicrobial activity against competing microorganisms such as bacteria, viruses, protozoa, filamentous fungi and yeasts (Niyonsaba et al., 2009; Tam et al., 2015). In plants AMPs can play a role in constitutive immunity or can be induced upon pathogen attack. Inducible responses can include the expression of pathogen-related (PR) proteins such as the enzymes (1-3)-β-glucanases (PR-2), chitinases (PR-3, -4, -8, and -11), peroxidases (PR-9) and oxalate oxidases (PR-16 and -17; Thatcher et al., 2005; Tam et al., 2015). In addition, it has been proposed that proteins involved in the delivery of storage and energy requirements to plant embryos during germination may also be involved in defense responses (Chrispeels and Raikhel, 1991; Marcus et al., 1999; Gábrišová et al., 2016). For example, members of the following storage protein families 2S albumins, Kunitz proteinase inhibitors, plant lectins and vicilins or vicilin-like proteins (including 7S globulins and β-conglutins; De Souza Candido et al., 2011).

Plant storage proteins can be classified into vegetative storage proteins and seed storage proteins, where the latter can represent a significant proportion of seed composition (Gomes et al., 2014). Storage proteins perform essential roles in plant survival. They provide a source of amino acids that can be mobilized and utilized for maintenance and growth during both seed embryonic developmental, and germination stages (Zienkiewicz et al., 2011; Tan-Wilson and Wilson, 2012; Jimenez-Lopez et al., 2016). These proteins accumulate in cellular storage vacuoles of seeds, nuts, and kernels; stem parenchyma of trees; grains and legumes; and some roots and tubers. The vicilins, also called conglutins in some legume species, constitute a class of proteins abundantly found as reserves in seeds of leguminous and non-leguminous plants, representing as much as 70 to 80% of total protein in the seeds of these plants (Duranti and Gius, 1997). Their structure consists of a trimeric organization, and unlike most plant storage proteins individual subunits with molecular masses typically around 15–70 kDa (Melo et al., 1994), NLL’s individual subunits are larger and range from 150 to 170 kDa in size (Argos et al., 1985).

Vicilins appear to play multifunctional roles, acting as an energy source and providing amino acids during the germination process, while in some cases, also being involved in defense responses against fungi and insects (Yunes et al., 1998). This includes for example, vicilins from the legumes *Vigna unguiculata* (cowpea), *V. radiata* (mung bean), *Phaseolus vulgaris* (common bean) and *Canavalia ensiformis* (jack bean; Gomes et al., 1997, 1998; Oliveira et al., 1999; Coda et al., 2008). The insecticidal activity of vicilins relates to their capacity to bind chitinous structures, thereby interfering with insect development, as shown for cowpea and the cowpea seed beetle (*Callosobruchus maculatus*; Sales et al., 2001). This chitin-binding activity can also inhibit yeast and fungal growth (Gomes et al., 1998). The potency of vicilin antifungal activity varies among plant species. For example, Gomes et al. (Gomes et al., 1998) extracted a vicilin from *V. unguiculata* showing inhibitory activity between 90 and 100% against the yeast *S. cerevisiae*, in addition to interfering with spore germination of the fungi *Fusarium solani, F. oxysporum, Colletotrichum musae, Phytophthora capsici, Neurospora crassa* and *Ustilago maydis sporidia*. Vicilin extracted from *V. radiata* seeds showed 65% inhibitory activity against *Candida albicans* (Gomes et al., 1998), whereas vicilin isolated from the non-legume *Malva parviflora* Malva (an annual or perennial herb) showed inhibitory activity against *Phytophthora infestans* (Wang et al., 2001).

Narrow-leafed lupin (*Lupinus angustifolius* L.; NLL) is a recently domesticated important pulse crop, and increasingly popular due to its wide range of agricultural and health benefits (Berger et al., 2013). The NLL grain constitutes an important source of protein for humans and animals with low starch content and free of gluten (reviewed in Foley et al., 2011). In NLL the seed storage proteins are collectively called conglutins and fall into four sub-families called α, β, γ, and δ-conglutins (Foley et al., 2011, 2015). In addition to dissection of lupin-based health benefits, the identification of lupin seed storage proteins playing roles in resistance against pathogens is of interest. Recently, antifungal activity from a multifunctional glyco-oligomer with 210 kDa, mainly composed by BLAD (*banda de Lupinus albus doce*), a 20 kDa polypeptide, a stable intermediary product of β-conglutin catabolism, was demonstrated and found to exclusively accumulate in the cotyledons of *Lupinus* species (Monteiro et al., 2015).

The recent development of a reference NLL genome assembly (Hane et al., 2016)^1^ and extensive RNA expression analysis from various tissues including seeds (Foley et al., 2015; Kamphuis et al., 2015) facilitated the identification of 16 *conglutin* genes, where the β*-conglutin* family was the most abundant, representing 56% of the total seed storage protein RNA expression levels (Foley et al., 2011). The NLL β-conglutin family comprises seven members, namely β1- to β7-conglutin (Foley et al., 2011). These β-conglutins share sequence identities ranging from 77.4 to 94.7%, reflected presumably in differential structure-functionality between some of them (Jin et al., 2014), and are highly expressed in the seeds compared to other NLL tissues (Foley et al., 2015).

Pathogenic fungi of lupins, as is the case for many other grain legume crops, cause substantial annual crop losses and are of major economic significance (Rubiales et al., 2015). For example, *Sclerotinia* stem rot, *Rhizoctonia* barepatch, *Phytophthora* root rot, and anthracnose stem and pod blights caused by *Colletotrichum lupini* causes several million dollars of losses in Australia, the largest producer of NLL globally (Sinden et al., 2004; Murray and Brennan, 2012). Considering the demonstrated antifungal activity of some seed storage proteins from several legume species, it was of interest to determine if seed storage proteins such as β-conglutins from NLL may also have roles in protection against fungal pathogens. Therefore the antifungal activity of NLL β-conglutins was examined using both *in vitro* and *in planta* assays for protection against fungal and oomycete pathogen growth known to induce necrotic host tissue damage. Furthermore, insight into the potential inhibitory mechanisms by which these proteins act against pathogens was obtained through an assessment of their subcellular localization and impact on plant oxidative processes.

## Materials and Methods

### Plant Material and Growth Conditions

Plant experiments were conducted with *Nicotiana benthamiana* accession “lab” (Bally et al., 2015) in temperature controlled growth rooms as described by Petrie et al. (2010). Plants were grown under a 16-h light/8-h dark cycle at 22°C.

### Fungal Isolates

Details of fungal isolates are listed in **Table 1** and were maintained as pure cultures with *Rhizoctonia solani, Alternaria brassicicola, F. oxysporum, Phytophthora nicotianae* and *C. lupini* isolates grown on 1/2 strength Potato Dextrose Agar (PDA), and *S. sclerotiorum* on 1/8 strength PDA. Spores, mycelia or sclerotia were inoculated on PDA plates, which were placed at room temperature in the dark until plates were fully covered by the pathogen. Mycelial plugs from these plates were used for subsequent experiments.

**Table 1:** Details of fungal isolates used to assess β-conglutin antifungal activity.

Fungal pathogen	Isolate	Host(s)	Isolated off	Reference
*Rhizoctonia solani* AG8-1	WAC10335	Lupin, cereals, Brassicas	Lupin	Perl-Treves et al., 2004; Hane et al., 2014
*Rhizoctonia solani* AG2-1	WAC9767	Lupin, Brassicas	Lupin	Perl-Treves et al., 2004
*F. oxysporum* f. sp. *medicaginis*	*Fom*-5190a	*Medicago* species	Alfalfa	Williams et al., 2016
*F. oxysporum* f. sp. *conglutinans*	*Fo*5176	Brassicas	Cabbage	Thatcher et al., 2012
*Alternaria brassicicola*	UQ4273	Brassicas	Cabbage	Schenk et al., 2000
*Sclerotinia sclerotiorum*	UQ3833	Dicots, broad host range	Canola	Supplied by Kemal Kazan, CSIRO
*Colletotrichum lupini*	WAC8672	Lupins	NLL	Supplied by Julie McClements, DAFWA
*Colletotrichum lupini*	WAC10444	Lupins	NLL	Supplied by Julie McClements, DAFWA
*Phytophthora nicotianae*	PAB12.23	*Solanaceae* family	Tobacco	Supplied by Giles Hardy, Murdoch University.

### Construction of Expression Plasmids

β1- and β6-conglutins were overexpressed using the pET28b construct (Novogen)^2^ that contains an N-terminal polyhistidine (6xHis) tag. pUC57 vectors carrying synthesized β1, β2, β3, β4, or β6 conglutin sequences based on Genbank HQ670409 (β1), HQ670410 (β2), HQ670411 (β3), HQ670412 (β4) and HQ670414 (β6) sequences but altered for optimum bacterial codon usage with *NcoI/XhoI* restriction enzyme linkers were synthesized and constructed by GenScript^3^ (**Supplementary Figure S1**). The bacterial expression vectors for β-conglutins were obtained via *NcoI/XhoI* digestion of respective pUC57-β-conglutin constructs followed by ligation of the β-conglutin fragments into the pET28b vector.

### Overexpression and Purification of NLL β-Conglutin Proteins

All β-conglutin proteins were expressed in Rosetta^TM^ 2(DE3) pLysS Singles^TM^ Competent Cells (Novogen). Protein expression was performed using an auto-induction method (Studier, 2005). Briefly, a single clone containing the expression construct was isolated and grown for 20 h in LB plus kanamycin at 50 μg/mL at 37°C and continuous shaking (200 rpm). The culture was diluted 1:150 in ZYM-5052 medium and grown for a further 5 h until the cell density reached an OD_600_ of 0.7. The cells were then induced to overexpress the proteins by adjusting the temperature to 19°C for another 20 h. Cells were collected by centrifugation at 5000 × *g* at 4°C. The bacterial cell pellet was rinsed two times with phosphate buffered saline (PBS), pH 7.5, removing the supernatant, then flash frozen in liquid nitrogen and stored at -80°C until further use.

### Purification of Recombinant β1- and β6-Conglutin Proteins

Protein purification from bacterial pellets was performed following the manufacturers’ recommendations for His-tagged proteins (Qiagen)^4^. Briefly, the steps consisted of lysing cells followed by nickel affinity chromatography using Ni-NTA spin columns, and histidine (6xHis) tags at the N-terminal part of the β-conglutin proteins. After elution of 6xHis-tagged proteins from the column with an increasing imidazole concentration gradient (10–300 mM), 2.5 mL fractions were collected. Fractions containing protein were analyzed using SDS-PAGE and fractions showing a single band corresponding to the expected molecular weight were pooled, and dialyzed five times against Tris-HCl 100 mM, pH 7.5, 150 mM NaCl to eliminate the imidazole reagent. The protein was concentrated using a 30 kDa Amicon centrifuge filter (Millipore)^5^. The aliquots were flash-frozen in liquid nitrogen and kept at -80°C until further use. Protein purities were >95% as determined by densitometry analysis of the SDS-PAGE gel image. An aliquot of each protein was used to measure their concentration using Bradford assays (BioRad, Hercules, CA, USA) using bovine serum albumin (BSA) as a standard. The β-conglutins purifications yields ranged between 10–15 mg/mL.

### β-Conglutin Antibody Production

The peptide sequence Nt – VDEGEGNYELVGIR – Ct, was chosen as this region was 100% homologous among all NLL β-conglutins and did not share any significant homology to other known lupin sequences. This peptide was generated by Agrisera^6^ and was used to immunize rabbits and to produce polyclonal antiserum (Agrisera). The rabbit immune serum was affinity-purified against the same synthetic peptide.

### SDS-PAGE and Immunoblotting

SDS-PAGE and Immunoblotting were performed as previously described (Foley et al., 2015).

### *In vitro* Assays for Fungal Growth Inhibition

A disk diffusion method was performed on 90 mm petri dishes containing PDA to test the sensitivity of different fungi strains toward the β-conglutin proteins. Fungal isolates were initially grown on PDA plates as described previously at 21°C until mycelial growth had developed. A mycelial plug was then taken from the growing edge of the colony and placed in the center of a new full-strength PDA plate in which sterile blank paper disks (12.7 mm diameter) containing 800 μg of purified β-conglutin protein (dissolved in BSA buffer) or buffer only control were placed 30 mm away. For *in vitro* assay of *C. lupini*, 1 mg of purified β6-conglutin protein was used. The plates were incubated in the dark at 21°C and the zone of fungal inhibition around the disks recorded over 30 days. Assays were performed in triplicate.

Antifungal activity of β-conglutin proteins were expressed as the IC50 (μM) values for the fungi tested in **Table 1**. The mycelial growth inhibition assays were used to determine the concentration required for 50% growth inhibition (IC50), using a β-conglutin protein concentration range from 5 to 125 μM (5, 10, 15, 20, 25, 35, 50, 75, and 125 μM) for each β-conglutin. Results were expressed as mean ± standard deviation (SD). To determine the statistical significant differences of the β-conglutins antifungal activity on the growth of these fungal pathogens, the data was analyzed using statistical package SPSS 15.0 (SPSS, Inc., Chicago, IL, USA). Significant differences between the mean values of each cohort were determined using Tukey Kramer HSD test (*p* < 0.05). To characterize the antifungal activity of β-conglutins further, a subsequent experiment with the necrotrophic fungal pathogen of NLL causing anthracnose disease (*C. lupini* isolates WAC8672 and WAC10444) was conducted, where the antifungal activity of β6-conglutin was determined by placing a mycelial plug in the center of a PDA plate and radial outgrowth determined over 14 days. For each time point significant differences were determined using a student’s *t*-test using the JMP software v7.0 (SAS Institute).

### Agroinfiltration of *Nicotiana benthamiana* Leaves

Four-week-old *N. benthamiana* plants grown at 22–24°C in culture rooms were used for *Agrobacterium tumefaciens*-mediated transient expression as described previously (Sparkes et al., 2006). The β1- and β6-conglutin coding sequence were cloned into the vector pMDC83 to generate C-terminal GFP fusion proteins and transformed into *A. tumefaciens* AGL1. Transformed *A. tumefaciens* AGL1 were cultured at 28°C until stationary phase (∼24 h), washed and resuspended in infiltration medium (50 mM MES, 0.5% (w/v) glucose, 100 μM acetosyringone (Sigma-Aldrich^7^ pH 5.6). The bacterial suspension was inoculated using a 1-mL syringe without a needle by gentle pressure through a <1 mm hole punched on the lower epidermal surface of the upper leaves of *N. benthamiana* plants. Following infiltration, plants were incubated under normal growth conditions at 22–24°C. This protocol was used for *in vivo* fungal growth inhibition assays, oxyblot assays, and subcellular localization studies.

### Trypan Blue Staining for Fungal Hyphae and Dead Plant Cells

Forty hours after *Agrobacterium* infiltration of β1- or β6-conglutin protein constructs into *N. benthamiana* plants, agar plugs containing hyphae of *S. sclerotiorum* or *P. nicotianae* were placed on the infiltrated leaf areas (control and β1- or β6-conlgutin overexpression) and plants were incubated in a growth chamber at 18°C for 3 days under a 16 h long-day light regime. Leaves were assessed for visible necrotic disease progression, then detached and further visualized after lactophenol trypan blue staining based on Keogh et al. (1980). Briefly, mature fourth leaves of *N. benthamiana* containing visible infection sites (tissue necrosis) were cleared with acetic acid: ethanol (1:1 v/v) then stained for 1–2 h in lactophenol (10 mL phenol; 10 mL lactic acid, 10 mL water) with 0.05% (w/v) trypan blue at 60°C. Excess staining was removed with lactic acid: water (1:1 v/v) until leaves were clear. Leaves were examined by light microscopy on a Nikon N400-M light microscope (Nikon, Tokyo, Japan). Control areas were infiltrated with GFP-only vectors. The experiment was repeated three times. In each experiment, leaves from 4–6 plants were analyzed for each treatment.

### Subcellular Localization of β1- and β6-Conglutin in Plant Cells

Fusion proteins were expressed in 3-week-old *N. benthamiana via Agrobacterium* infiltration of leaves as previously described (Sparkes et al., 2006). Leaves were excised 3 days following infiltration and mounted with water under a 0.17 mm coverslip and imaged using a Nikon A1Si confocal microscope (Nikon Plan Apo VC 60x NA1.2 water-immersion objective). For GFP imaging, the 488 nm laser line and a 521/50 nm band pass filter was utilized, while a 561 nm laser line and 595/50 nm filter was used for RFP imaging.

Images were analyzed using ImageJ software (Schneider et al., 2012). Images were converted to 8-bit grayscale and the intensity correlation analysis (ICA) method was used for determine the levels of colocalization and by using the JACoP plugin according to (Bolte and Cordelières, 2006). As a control, empty vector was used to transform leaf cells expressing 35S::GFP alone as described previously by Thatcher et al. (2007).

### Oxyblot Assays

Proteins were extracted from *N. bentamiana* leaves infiltrated with GFP, β1-GFP or β6-GFP fusion expression constructs following either control or *S. sclerotiorum* or *P. nicotianae* treatments [extraction buffer: 25 mM Tris–HCl, pH 7.0, 0.05% Triton X-100, 1 mM dithiothreitol (DTT), and protease inhibitors (Roche, Basel, Switzerland)]. 25 μg of total proteins were loaded onto 12% polyacrylamide gels for protein separation. Proteins separated by SDS-PAGE were electrotransferred to PVDF membranes. The OxyBlot^TM^ Protein Oxidation Detection Kit (EMD Millipore) was used according to the manufacturer’s instructions for immunoblot detection of carbonyl groups introduced into proteins by reaction with reactive oxygen species (ROS).

## Results

### *In vitro* Inhibition of Fungal Growth by β-Conglutin Recombinant Proteins

To assess the potential for antifungal activity in NLL seed storage proteins, we focussed on the most abundant seed storage proteins in the NLL grain, the β-conglutins (Foley et al., 2011). The 6xHis-tag recombinant β-conglutin proteins were expressed in *E. coli* and purified using nickel affinity chromatography. To confirm the identity of purified β-conglutins, SDS–PAGE analysis of the purified proteins was performed, which indicated a single protein band of approximately 65 kDa, which is the predicted size of β-conglutin (**Supplementary Figure S2A**). This was followed by immunoblotting using an anti-β-conglutin antibody which confirmed the identity of the recombinant proteins as β-conglutin (**Supplementary Figure S2B**). We were successful in expressing and purifying the β1, β2, β3, β4 and β6 recombinant proteins but not β5 and β7.

Subsequently, we examined the effect of the purified β-conglutin proteins on the growth rate of a range of phytopathogenic necrotrophic fungi using *in vitro* bioassays. The fungal pathogens selected included the legume pathogen *F. oxysporum* forma specialis (f. sp.) *medicaginis* (*Fom*-5190a, a root pathogen), the broad host range pathogens *R. solani* AG8-1 (isolated from lupin) and *S. sclerotiorum* (isolated from canola) and the brassica-specific pathogens *F. oxysporum* f. sp. *conglutinans* (*Fo*-5176), *R. solani* AG2-1 and *A. brassicicola* (*Brassicaceae* hosts; details of these pathogens are listed in **Table 1**). The IC50 values (μM) for each of these fungal isolates was determined (**Table 2**). Overall the β1 and β6 conglutins showed significantly stronger mycelium growth inhibition when compared to β2-, β3-, and β4-conglutin proteins for both *R. solani* isolates and *A. brassicicola* by Tukey–Kramer honestly significant difference (HSD) test (*P* < 0.05). The β1-conglutin showed a significantly stronger growth inhibition to *S. sclerotiorum* and the two *F. oxysporum* isolates, compared to β2-, β3-, and β4-conglutin, where β6-conglutin was not significantly different from β1 (**Table 2**). Overall β1-, and β6-conglutin showed the strongest mycelial growth inhibition to the various pathogens tested, but interestingly, a sequence alignment of the seven β-conglutin proteins showed β6 exhibits the highest sequence identity to other β-conglutin protein isoforms (78–98%) while β1 had an amino acid sequence with the lowest identity (77–81%; **Supplementary Figure S3**). Control treatment of filter disks with BSA buffer showed no fungal growth inhibition against any of the isolates tested.

**Table 2:** Antifungal activity of the NLL recombinant purified β1- to β4-conglutins and β6-conglutins.

	IC50 values (μM)					
Fungi species	Isolate	β1	β2	β3	β4	β6
*Rhizoctonia solani* AG8-1	WAC10335	18.5 ± 2.5^c^	43.0 ± 4.0^ab^	36.8 ± 3.7^b^	52.5 ± 5.5^a^	20.5 ± 2.0^c^
*Rhizoctonia solani* AG2-1	WAC9767	17.4 ± 2.0^c^	45.2 ± 3.5^a^	31.5 ± 3.8^b^	46.2 ± 4.1^a^	23.2 ± 1.7^c^
*Sclerotinia sclerotiorum*	UQ3833	14.0 ± 1.3^c^	27.0 ± 4.2^a^	23.6 ± 2.0^ab^	26.3 ± 3.2^ab^	19.3 ± 2.4^bc^
*Alternaria brassicicola*	UQ4273	16.0 ± 1.7^b^	34.0 ± 4.6^a^	24.0 ± 3.3^b^	37.8 ± 3.0^a^	21.6 ± 2.2^b^
*F. oxysporum f. sp. conglutinans*	*Fo*5176	18.7 ± 1.5^c^	41.3 ± 5.8^a^	29.7 ± 2.5^b^	40.1 ± 3.8^a^	25.5 ± 3.5^bc^
*F. oxysporum f. sp. medicaginis*	*Fom*-5190a	20.3 ± 2.7^c^	39.0 ± 5.2^ab^	32.0 ± 4.2^b^	44.3 ± 5.0^a^	29.7 ± 4.0^bc^

*Protein concentrations (μM) required for IC50 were determined from the dose- response curves (percentage of growth inhibition versus protein concentration). The results are expressed as mean ± standard deviation (SD) of three biological replicates. Statistically significant differences were calculated using a Tukey–Kramer honestly significant difference (HSD) test (*P* < 0.05). Different letters for each of the different pathogens indicate significant difference in IC50 value of the β-conglutin.*

**FIGURE 1:**
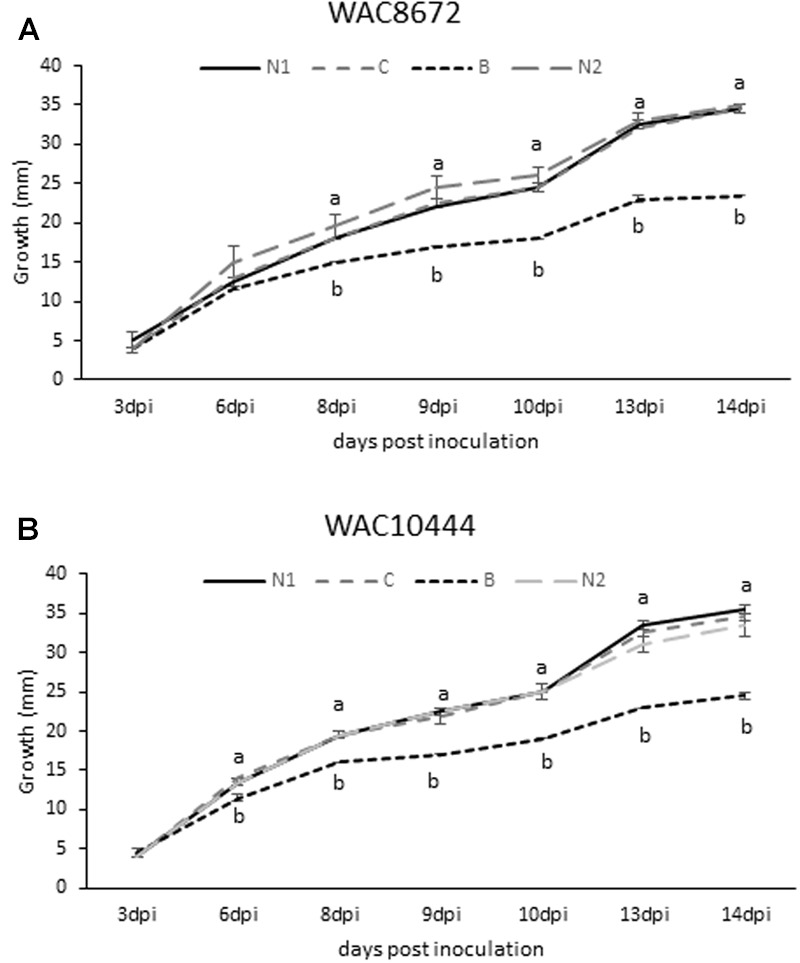
**Antifungal activity of recombinant β6-conglutin in *in vitro* bioassays against the lupin pathogen *Colletotrichum lupini*.** Significant growth inhibition toward β6-conglutin was observed from as early as **(A)** 8 dpi for isolate WAC8672 and **(B)** 6 dpi for isolate WAC10444. Means and standard error of three biological replicates. Letters indicate significant differences for a given day after inoculation (dpi) with the mycelial plug by student’s *t-*test. **C**, control containing 1 mg BSA; **N1**, no protein; **B**, 1 mg β6-conglutin; **N2**, no protein.

Based on the β-conglutin protein alignments and IC50 data, we decided to focus on β6-conglutin as a representative member of the β-conglutin family. Further characterization of the *in vitro* anti-fungal properties of β6-conglutin was performed in a detailed time course experiment against two isolates (WAC8672 and WAC10444) of a major fungal pathogen of lupins, *C. lupini* which causes anthracnose disease (Fischer et al., 2015). Radial outgrowth of *C. lupini* mycelium on PDA plates toward Whatman filter disks containing control protein (BSA), β6-conglutin or no protein was recorded. Radial growth inhibition was only observed toward filter disks containing β6-conglutin protein and this occurred from as early as 6 days post-inoculation with the mycelial plug for isolate WAC10444 and 8 days post-inoculation for WAC8672 (**Figure 1**). Combined with the IC50 data in **Table 2**, these results indicate recombinant NLL β-conglutins exhibit antifungal activity *in vitro* against both leaf and root-infecting pathogens of legumes and non-legume hosts.

### β1- and β6-Conglutins Exhibit *in planta* Anti-fungal and Oomycete Activity

**FIGURE 2:**
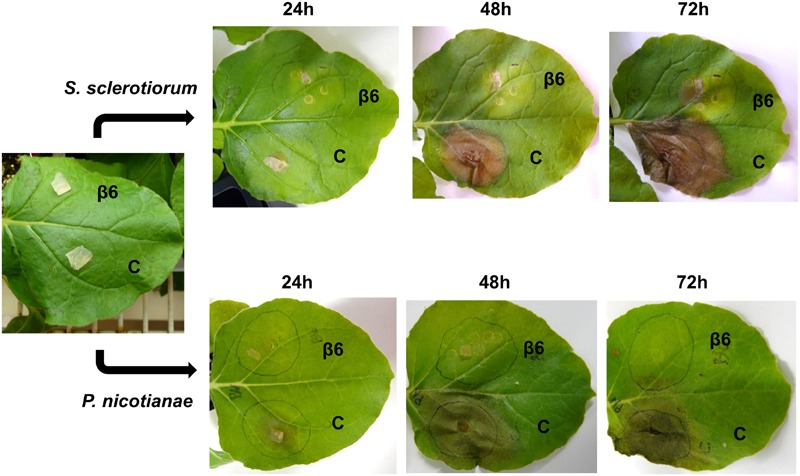
**Recombinant β6-conglutin exhibits *in planta* anti-fungal and oomycete activity.** Shown are representative images of *Agrobacterium* infiltrated *N. benthamiana* leaves expressing recombinant β6-conglutin proteins and subsequently inoculated with either *S. sclerotiorum* or *P. nicotianae*. The experiment was repeated three times with similar results. C, control agroinfiltration of the leaf area with *Agrobacterium* expressing GFP only; β6, agroinfiltration of the leaf area with *Agrobacterium* expressing GFP tagged β6-conglutin.

To examine the effect of NLL β6-conglutin *in planta*, we selected the *N. benthamiana* infiltration system as a model for assessing the functionality of proteins against various phytopathogens (Ma et al., 2012). This involved *Agrobacterium*-mediated infiltration into leaves of *N. benthamiana* plants followed by assessment of antifungal activity in disease assays. The broad host range leaf pathogen *S. sclerotiorum* was chosen which is readily amenable to *N. benthamiana* leaf disease assays and secretes the non-host selective toxin oxalic acid to induce disease symptom development (Kim et al., 2008; Williams et al., 2011). In addition, β6-conglutin showed strong inhibition of this pathogen’s growth as compared to other fungal pathogens tested in our *in vitro* assays (**Table 2**). The *Agrobacterium*-infiltration system was used to transiently express β6-GFP or a GFP-only control. The GFP control was infiltrated into one half of the leaf with β6-GFP infiltrated into the other leaf half. Forty-eight hours after infiltration, leaves were inoculated with *S. sclerotiorum* and progression of lesions was observed over 72 h (**Figure 2**). In the GFP-only control, necrosis was apparent within 24 h of *S. sclerotiorum* inoculation with the size of the necrotic lesions progressing rapidly to engulf half of the leaf by 72 h. In stark contrast, there was only limited necrotic damage in the leaves expressing the β6-GFP protein. As β1-conglutin demonstrated strong antifungal activity *in vitro* yet exhibits the least amino acid identity amongst the NLL β-conglutins, we also assayed β1-GFP in the above experiments. As with β6-GFP, β1-GFP infiltrated leaves also exhibited limited necrotic damage after *S. sclerotiorum* inoculation (**Supplementary Figure S4**).

The soil-borne oomycete pathogen of *N. benthamiana, P. nicotianae*, was also assayed. *P. nicotianae* is a hemibiotrophic pathogen that causes root rot, leaf necrosis and stem lesions (Liu et al., 2016). As with the *S. sclerotiorum* assays, both β6- and β1-conglutin strongly inhibited lesion development by *P. nicotianae* in our *N. benthamiana Agrobacterium*-infiltration disease assays (**Figure 2** and **Supplementary Figure S4**). At 72 h post *S. sclerotiorum* or *P. nicotianae* inoculation, β6-conglutin infiltrated leaf zones exhibited a 71.7 and 85.7% reduction in lesion size, respectively, relative to control treated leaf zones. Similar reductions were recorded for β1-conglutin infiltrated leaf zones (94.2 and 90.3%).

### β1- and β6-Conglutin Reduce Pathogen Growth and Pathogen Induced Cell Death *In planta*

**FIGURE 3:**
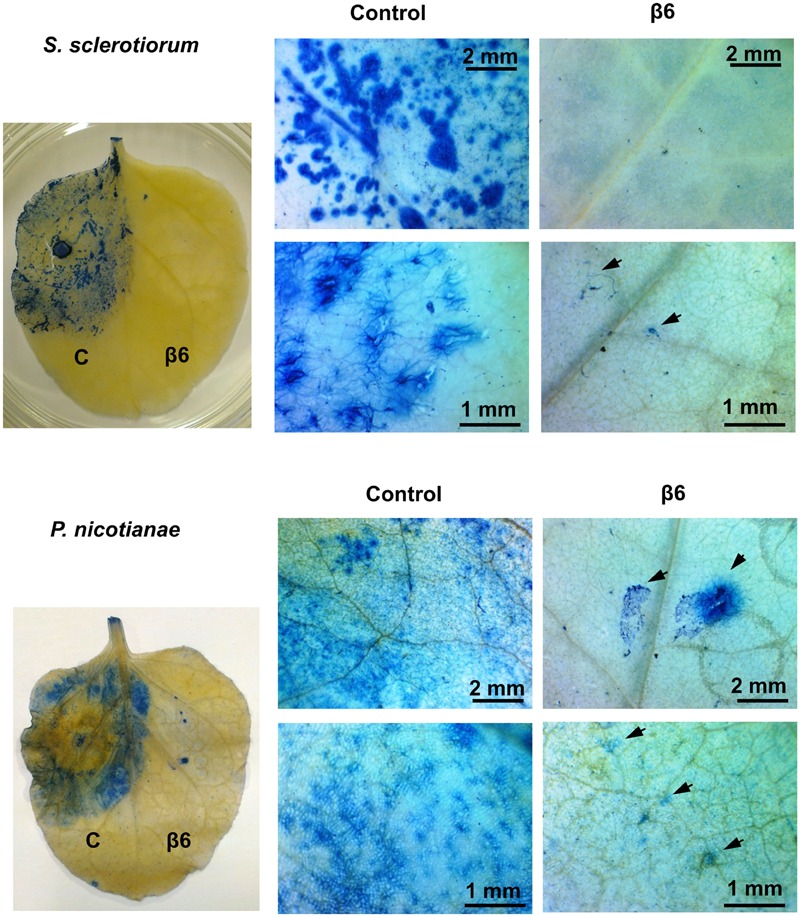
**Recombinant β6-conglutin reduces pathogen growth and pathogen induced cell death *in planta*.** Shown are representative images of *Agrobacterium* infiltrated *N. benthamiana* leaves expressing recombinant β6-conglutin proteins and subsequently inoculated either *S. sclerotiorum or P. nicotianae*. Trypan blue staining was performed to visualize hyphal growth and cell death. Arrows point to hyphae and hyphal damage.

The striking inhibition of *S. sclerotiorum* and *P. nicotianae* induced lesions on *N. benthamiana* leaves expressing β1- or β6-conglutin suggests these pathogens are unable to grow or their growth is severely impaired by these β-conglutins. To examine fungal/oomycete growth we challenged *N. benthamiana* β-conglutin expressing leaves with pathogen and allowed 72 h for disease symptom development in controls, then assessed the leaves and mycelial growth microscopically after staining with trypan blue, which stains dead plant cells (van Wees, 2008). In leaves expressing β6-GFP or β1-GFP we observed strong inhibition of the *S. sclerotiorum* and *P. nicotianae* mycelial growth compared to controls (**Figure 3** and **Supplementary Figure S5**). There was evidence of aggregated cells with short pseudohyphae, particularly at the site of inoculation, however, these were few and sparse in comparison to controls. Combined, our results suggest β1- and β6-conglutins from NLL inhibit hyphal growth of a range of phytopathogenic oomycete and fungi, both *in vitro* and *in vivo.*

### Subcellular Localization of β-Conglutin in *N. benthamiana* Leaves

**FIGURE 4:**
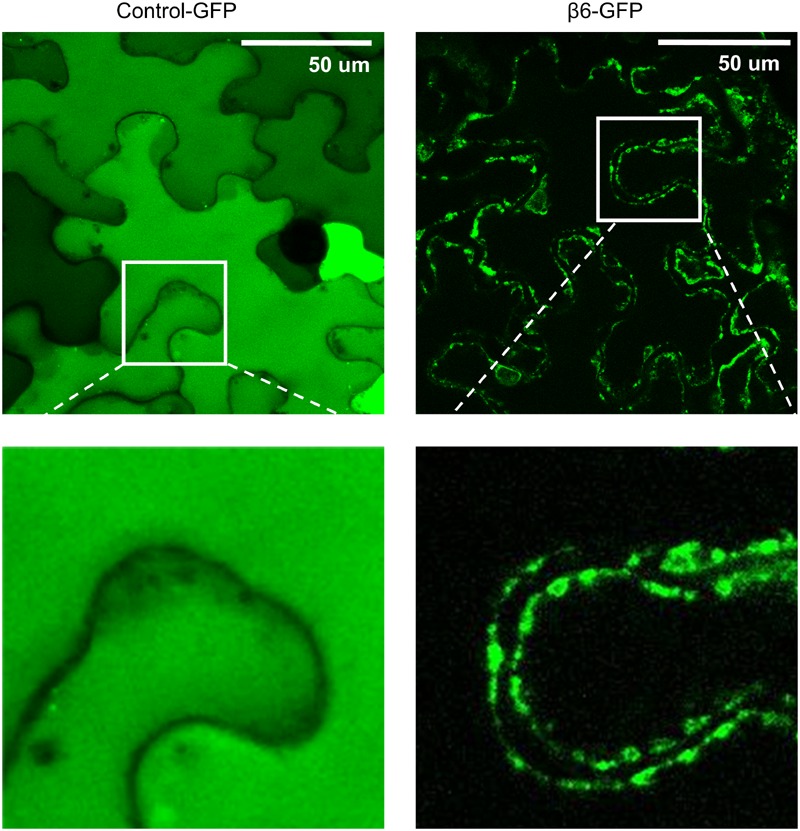
**β6-conglutin is localized to the cell surface.** Confocal images of tobacco epidermis cell expressing GFP alone or β6-GFP shows GFP alone homogenously expressed throughout the cytoplasm while β6-conglutin localizes to the plasma membrane through the whole cell, with no expression in cytosol or intracellular organelles. Insert: β6-GFP shows punctate labeling at the cell surface.

**FIGURE 5:**
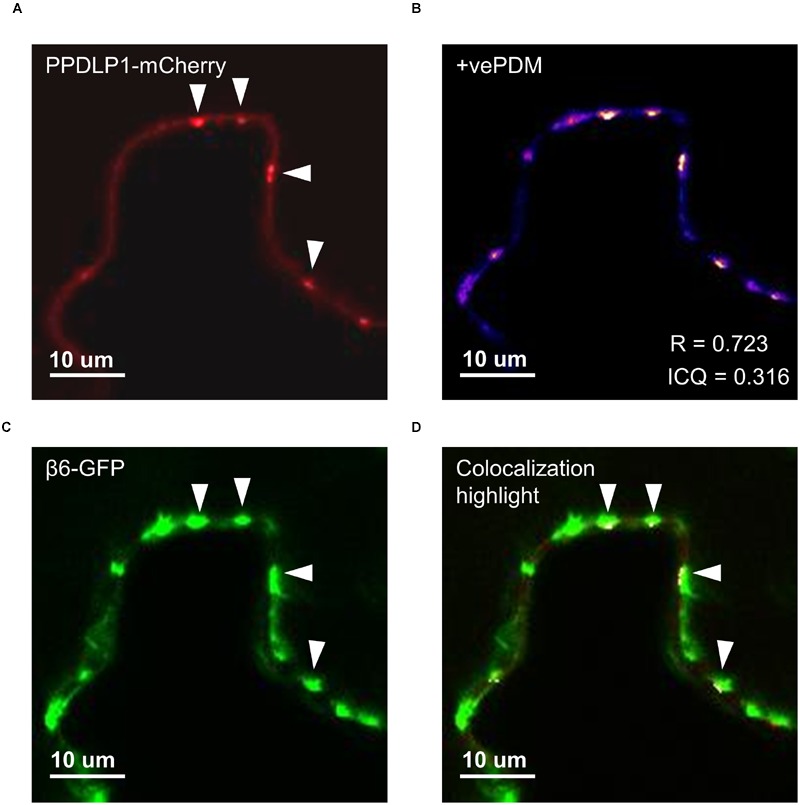
**β6-conglutin localizes to the plasmodesmata.** Single-slice confocal images of co-expression GFP-β6 with the plasmodesmata marker PDLP1-mCherry after transient expression in *N. benthamiana*; **(A)** PPDLP1-mCherry, **(B)** β6-GFP, **(C)** Image showing pixel pairs that have a positive PDM value equal to the value (intensity of A- mean A intensity) ^∗^ (intensity of B-mean B intensity) as described in Li et al. (2004), **(D)** merge of **(A,B)** with highlighted co-localized pixels. ICQ, Intensity correlation quotient; R, Mandel’s overlap coefficient. 60× immersion objective.

The effect of β-conglutins on the growth of fungal and oomycete pathogens tested led to the hypothesis that β1- and β6-conglutins might localize at the cell surface, at sites closest to initial pathogen attack. Therefore, the subcellular localization of the β-conglutin proteins using *Agrobacterium*-mediated transient expression of β-GFP constructs in *N. benthamiana* leaves was examined. Two-to-three days following *Agrobacterium* infiltration, the localisation of β-GFP in *N. benthamiana* leaf epidermal cells was examined by confocal microscopy. Both β6-GFP and β1-GFP were expressed in punctate structures close to the cell surface (plasma membrane; **Figure 4, Supplementary Figure S6**). To determine the nature of the β6-GFP- and β1-GFP-positive structures, which resembled the pattern described for plasmodesmata (Lee and Lu, 2011), we co-expressed the constructs with the known plasmodesmata marker plasmodesmata-located protein1 or AtPDLP1-mCherry (Thomas et al., 2008). β6-GFP and β1-GFP partially overlapped the expression pattern of AtPDLP1-mCherry (*R* = 0.0725–0.755), indicating that β6-GFP and β1-GFP were partially located at plasmodesmata (**Figure 5**, and **Supplementary Figure S6**).

### Protein Oxidation Levels in *N. benthamiana* Leaves Expressing the β1- and β6-Conglutin Proteins Following Infection with *S. sclerotiorum* and *P. nicotianae*

**FIGURE 6:**
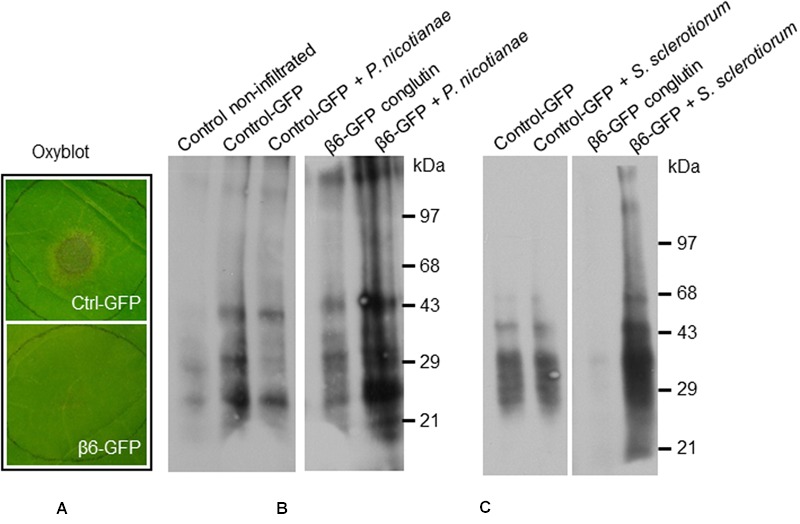
**β6-conglutin oxyblots assayes.** Protein carbonyl formation in tobacco leaves 48 h after agroinfiltration with indicated constructs and 24 h after inoculation with pathogens. Protein carbonyls were assessed using an OxyBlot TM kit. **(A)** Typical levels of pathogen growth 24 h after inoculation of leaf samples used for assay. **(B)** Representative blot showing basal carbonylation levels in non-transformed leaves, control leaves expressing GFP mock-inoculated and after infection with *P. nicotianae*, and leaves expressing β6-GFP mock-inoculated and after infection. **(C)** Protein carbonylation levels after infection with *S. sclerotiorum*, same constructs as in **(B)**.

One of the mechanisms employed by *S. sclerotiorum and P. nicotianae* during infection of a compatible host is to initially suppress the plant second-phase oxidative burst that occurs 3–6 h after pathogen contact (Levine et al., 1994; Cessna et al., 2000), thereby compromising the capacity of the plant to activate downstream defense pathways (Doke, 1985; Levine et al., 1994; Williams et al., 2011). Therefore the effect of β-conglutin protein on the capacity of *S. sclerotiorum and P. nicotianae* to suppress the plant oxidative burst was examined. Leaves were infiltrated and allowed to transiently express GFP or β6-GFP conglutin for 48 h before being infected with *S. sclerotiorum or P. nicotianae*. As soon as hyphae and lesions became visible (within 24 h) leaves were collected for analysis (as shown in **Figure 6A**). We estimated production of ROS and oxidative burst capacity by examining the level of protein carbonylation in infected compared to mock-inoculated leaves using an OxyBlot Protein Oxidation Detection and immunoassay (Rinalducci et al., 2008). Protein oxidation is one of the covalent modification of proteins induced by ROS such as H_2_O_2_ or other products of oxidative stress, and carbonylation is one of the most commonly occurring oxidative modifications of proteins, which may be responsible for the alteration in protein activity, for example, signaling (Oracz et al., 2007). Carbonylated proteins have been identified in many plant species at different stage of growth and development (Barba-Espín et al., 2011; Morscher et al., 2015).

Basal levels of protein oxidation, as generated through normal metabolic activity (Alscher et al., 1997; Rinalducci et al., 2008) were observed in the mock-inoculated control leaves expressing GFP-only, as well as in mock-inoculated leaves expressing β6-GFP (**Figures 6B–C**). Following inoculation with *S. sclerotiorum* or *P. nicotianae* protein oxidation remained at similar levels in the GFP-only control leaves (**Figures 6B–C**). In contrast, we observed a marked increase in the levels of protein oxidation in leaves expressing the β6-GFP following infection with *S. sclerotiorum or P. nicotianae* when compared to the respective mock-inoculated β6-GFP or the infected GFP-only leaves. The inoculated leaves expressing β6-GFP were nevertheless healthy, as expected (**Figure 6A**). This suggests that the over-expression of β-conglutin proteins effectively circumvents the initial suppression of the plant oxidative burst by *S. sclerotiorum or P. nicotianae.*

## Discussion

β-conglutins are the most abundant seed storage proteins in NLL (Foley et al., 2011) and while in other plant species these vicilin-like proteins may have roles in plant defense, the functional roles of β-conglutins in this aspect remain largely unknown (Khuri et al., 2001; Dunwell et al., 2004). In this study we identified two NLL β-conglutin proteins that strongly inhibited the growth of a range of necrotrophic fungal or oomycete pathogens, both *in vitro* and *in vivo* when transiently expressed in *N. benthamiana* leaves. Reduced *in planta* fungal growth was associated with a significant reduction in pathogen-induced host cell death and interestingly the NLL β-conglutins examined were localized near the plant cell surface. These results provide the first demonstration for any NLL β-conglutin in protection against pathogen attack, and add to the growing list of vicilin-like proteins that accumulate during seed development and have roles in plant defense (Gomes et al., 1998; Marcus et al., 1999; Rietz et al., 2012; Monteiro et al., 2015).

Vicilin-like proteins are members of the cupin superfamily which is extremely diverse, encompassing 18 different functional classes including the vicilins and similar germin-like seed storage proteins, as well as single-barrel isomerases, epimerases, and auxin-binding proteins (Dunwell et al., 2001). Given the varying antifungal potency of vicilin and vicilin-like proteins from various plant species (Gomes et al., 1998), to determine and compare and contrast the ability of NLL β-conglutins to inhibit fungal growth we assayed each of the five synthesizable NLL β-conglutins against a range of necrotrophic pathogens. Of the five NLL β-conglutins, β1 and β6 exhibited the strongest activity *in vitro*. Sequence comparisons among the β-conglutins does not reveal any motif common between β1 and β6 but not in the other tested β-conglutins (**Supplementary Figure S3**) so at this stage we are unable to hypothesize why β1 and β6 have stronger fungal inhibition activity than the other β-conglutins. Furthermore, we demonstrated β-conglutin antifungal activity *in planta* against *S. sclerotiorum* as well as against a hemibiotrophic oomycete pathogen, *P. nicotianae*. Necrotrophic fungal pathogens actively kill host tissue, while hemibiotrophic pathogens switch to this attack mode during later stages of their infection cycle (Glazebrook, 2005).

Although plant defense responses against pathogen attack are the result of various integrated preformed and induced mechanisms, one of the most prominent is the hypersensitivity response (HR) resulting from the generation of host ROS (Mittler, 2002). While the HR response is a type of programmed cell death that can limit the growth of biotrophic pathogens, it is favorable to necrotrophic pathogens that thrive off the dead host cells (Glazebrook, 2005; Laluk and Mengiste, 2010). Both *S. sclerotiorum* and *P. nicotianae* are capable of inciting necrotic lesions on a broad range of host plants (Agrios, 2005; Gallup et al., 2006) where *S. sclerotiorum* produces the major pathogenicity factor oxalic acid (Cessna et al., 2000), the primary determinant contributing to its pathogenic success (Kim et al., 2011). In compatible interactions, oxalic acid initially dampens the plant oxidative burst (Williams et al., 2011). However, once the pathogen is established, oxalic acid induces apoptotic-like programmed cell death in plant hosts, triggered by the generation of ROS at detrimental levels (Kim et al., 2008). We found *in planta* expression of NLL β1- and β6-conglutins effectively impaired host cell death induced by both *S. sclerotiorum* and *P. nicotianae*, evident within 24 h of pathogen challenge and lasting over the 72 h assayed. *In planta* expression of NLL β1 and β6-conglutins also increased levels of pathogen (*S. sclerotiorum, P. nicotianae*) induced protein oxidation whilst maintaining leaf health, suggesting overexpression of these two β-conglutins inhibits pathogen induced suppression of the early phase plant oxidative burst.

To dissect how NLL β-conglutins inhibit pathogen growth and host cell death *in planta*, we utilized GFP-tagged versions of these proteins to visualize their sub-cellular localisation. The vacuolar localisation of vicilin-like proteins (to supply amino acids during seed germination and seedling growth) has been extensively reported, however, almost no studies have been conducted for these protein classes in organs other than seeds (Overvoorde et al., 1997). Germin-like proteins from peanut localize to both the cytoplasm and the cell surface (cell membrane or cell wall) when transiently expressed within onion epidermal cells (Wang et al., 2013). Here we observed both the NLL β1- and β6-conglutin proteins localizing to the cell surface in distinct structures that included plasmodesmata when expressed in *N. benthamiana* leaf epidermal cells. Many studies have demonstrated that ROS are produced at the plant cell wall in a highly regulated manner (Wojtaszek, 1997; Sewelam et al., 2016), where they play key signaling roles in the control of physiological processes such as cellular growth and development (Gapper and Dolan, 2006; Kärkönen and Kuchitsu, 2015), as well as adaptation to environmental changes and pathogen attack (Wu et al., 1997; Sewelam et al., 2016). In plants one of the major contributors to ROS production during pathogen infection are the plasma membrane localized NADPH oxidases (Torres et al., 2006; reviewed in Schopfer and Liszkay, 2006). It is possible that NLL β-conglutins facilitate/mediate the production of ROS directed to the oxidative burst (Bolwell et al., 1995; Bolwell and Wojtaszek, 1997), which is known to induce structural reinforcement of the cell wall through lignin crosslinking. This has been reported for some cupins (germin and germin-like proteins) from wheat (Schweizer et al., 1999). Alternatively, ROS such as H_2_O_2_ could play direct antimicrobial roles or act as a signaling molecule in defense response pathways (reviewed in Shetty et al., 2008).

Structurally, the β-conglutin proteins are similar to germins, germin-like proteins, and vicilin-like glucose binding proteins, which are also glycoproteins characterized by a beta-barrel core structure that can be associated with the cell wall (Lane et al., 1992), the plasma membrane (Overvoorde et al., 1997; Kukavica et al., 2005) and/or plasmodesmata (Ham et al., 2012). The structure of β-conglutin is unique as it possesses two cupin domains forming a Rossmann fold reminiscent of enzymes that use molecular oxygen as a substrate (Jimenez-Lopez et al., 2015). Germins and germin-like proteins have been shown to play dual roles in seed germination and also in pathogen defense (Cândido et al., 2011). Identified from germinating wheat embryos, the wheat germin protein exhibits oxalate oxidase activity, catalyzing the conversion of oxalates (the conjugate base of oxalic acid) into CO_2_ and H_2_O_2_ (Woo et al., 2000; Pan et al., 2007). Other enzymatic properties of germins or germin-like proteins include superoxide dismutase (SOD) activity, ADP glucose pyrophosphatase/phosphodiesterase activity or polyphenol oxidase (PPO) activity (reviewed in Barman and Banerjee, 2015). Over-expression of germin in several plant species can lead to increased resistance to fungal pathogens such as *S. sclerotiorum* (Donaldson et al., 2001; Dong et al., 2008; Walz et al., 2008), and the over-expression of a germin-like oxalate oxidase in rice or sunflowers lead to increased resistance, respectively, against *R. solani* (Molla et al., 2013) or both *R. solani* and *S. sclerotiorum* (Beracochea et al., 2015). Moreover, overexpression of the sunflower germin-like protein in Arabidopsis altered host redox and increased endogenous ROS levels (Beracochea et al., 2015). Germin-like proteins from *Brassica napus* have also been linked to the initiation of an oxidative burst that impedes pathogenesis of *S. sclerotiorum* (Rietz et al., 2012).

A role for β-conglutin in pathogen resistance has also been proposed based on cleavage and secretion of a β-conglutin peptide (BLAD) upon germination in *L. albus* (Monteiro et al., 2015). BLAD, a 20 kDa polypeptide, accumulates exclusively in the cotyledon between days 4 and 12 after the onset of germination. BLAD forms a 120 kDA oligomeric structure which exhibits lectin-like activity, catalytic activities of β-N-acetyl-D-glucosaminidase and chitin-binding activity, and provides effective antifungal activity against a range of plant pathogens (Monteiro et al., 2015). Whilst the results presented in our current study indicate the involvement of the NLL β-conglutin proteins in facilitating the production of ROS following pathogen infection *in planta*, it remains possible that some of the effects observed, particularly those obtained with the *in vitro* plate assays, may be partially linked to anti-fungal activities similar to those observed with the BLAD peptide. Protein exudates of germinating *L. albus* seeds showed fungal growth inhibition to five of six pathogens tested (Scarafoni et al., 2013). This protein exudate contains a range of different proteins including both β- and γ-conglutins. Our research presented herein has shown that β-conglutins have antifungal activity and the β-conglutins from *L. albus* in the protein exudate could thus be a good candidate for contributing to the causal antifungal activity observed. It is therefore possible that lupins secrete β-conglutins during the vulnerable initial seedling germination stage as a means to protect itself from plant pathogens. As the BLAD peptide has been processed from β-conglutin, it remains to be determined if β1 and β6 would have altered antifungal properties if these proteins were also processed similar to that of BLAD.

The results presented herein suggest that NLL β-conglutins may be more versatile in their physiological roles than previously thought. While a clear causal connection cannot be given at present, our results show that several NLL β-conglutins inhibit fungal growth *in vitro* and that expression of at least two of these *in planta* enhances plant resistance to fungal/oomycete necrotrophic pathogens.

## Author Contributions

Conceived and designed the experiments: JJ-L, SM, KD, and LT. Performed the experiments: JJ-L, SM, KD, LT, and LK. Analyzed the data: JJ-L, SM, KD, LT, LK, and KS. Contributed reagents/materials/analysis tools: RF, JJ-L, and KS. Wrote the paper: JJ-L, SM, KD, LT, RF, LK, and KS.

## Conflict of Interest Statement

The authors declare that the research was conducted in the absence of any commercial or financial relationships that could be construed as a potential conflict of interest.

The reviewer SF and handling Editor declared their shared affiliation, and the handling Editor states that the process nevertheless met the standards of a fair and objective review.

## Footnotes

1 http://www.lupinexpress.org/

2 www.novogen.com

3 www.genscript.com

4 www.qiagen.com

5 http://www.emdmillipore.com

6 http://www.agrisera.com/

7 www.sigmaaldrich.com

## Supplementary Material

FIGURE S1 **Sequences of synthetic β1, β2, β3, β4, and β6 conglutins that were cloned into the expression vector, pET28b**.

FIGURE S2 **Purification and confirmation of recombinant β1- to β4- and β6-conglutins. (A)** Purified proteins (10 μg per sample) were separated by SDS–PAGE analyses to indicate a single protein band (6xHis-tag) of approximately 65 kDa at high purity level (>95%). **(B)** Immunoblot confirmation using anti-β-conglutin protein antibody. The arrow indicates the correct sized band.

FIGURE S3 **Comparison of the NLL β-conglutin protein sequences.** Alignment of the seven β-conglutin proteins identified in NLL, and comparison of the level of variability among them. Similarity percentages for each compared-pair of sequences are described in the table below. The lowest and the highest percentages of similarity are highlighted with gray color.

FIGURE S4 **Recombinant β1-conglutin exhibits *in planta* anti-fungal and oomycete activity.** Shown are representative images of *Agrobacterium* infiltrated *N. benthamiana* leaves expressing recombinant β1-conglutin proteins and subsequently inoculated with either *S. sclerotorium* or *P. nicotianae*. The experiment was repeated three times with similar results.

FIGURE S5 **Recombinant β1-conglutin reduces pathogen growth and pathogen induced cell death *in planta*.** Shown are representative images of *Agrobacterium* infiltrated *N. benthamiana* leaves expressing recombinant β1-conglutin proteins and subsequently inoculated either *S. sclerotorium or P. nicotianae*. Trypan blue staining was performed to visualize hyphal growth and cell death. Arrows point to hyphae, asterisk marks inoculation site.

FIGURE S6 **β1-conglutin is localized to the cell surface and plasmodesmata. (A)** Confocal images of tobacco epidermis cell expressing GFP alone or β1-GFP. Insert: β1-GFP shows punctate labeling at the cell surface. **(B–E)** Single-slice confocal images of co-expression β1-GFP with the plasmodesmata marker PPDLP1-mCherry after transient expression in *N. benthamiana*; **(B)** PDLP1-mCherry, **(C)** β1-GFP, **(D)** Image showing pixel pairs that have a positive PDM value equal to the value (intensity of B- mean B intensity) ^∗^ (intensity of C-mean C intensity) as described in Li et al. (2004), **(E)** merge of **(B,C)** with highlighted co-localized pixels. **ICQ**, Intensity correlation quotient; **R** = Mandel’s overlap coefficient. 60× immersion objective.
