# Supplementary figures and images for: Narrow-Leafed Lupin (Lupinus angustifolius) β1- and β6-Conglutin Proteins Exhibit Antifungal Activity, Protecting Plants against Necrotrophic Pathogen Induced Damage from Sclerotinia sclerotiorum and Phytophthora nicotianae
Source: Front Plant Sci. 2016 Dec 9;7:1856. doi: 10.3389/fpls.2016.01856 (PMC5161055; doi:10.3389/fpls.2016.01856)

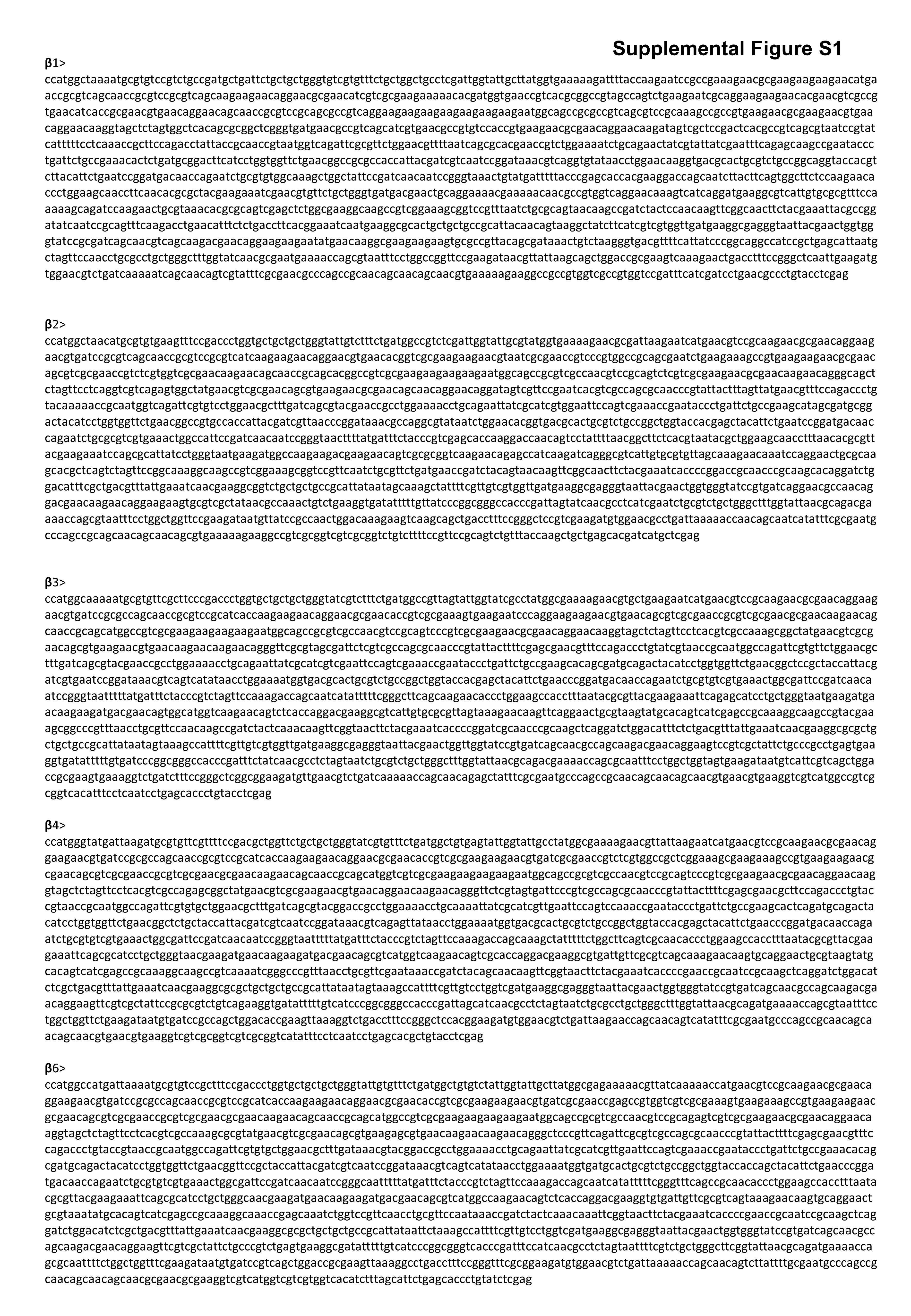

Supplement: FIGURE S1 — Sequences of synthetic β1, β2, β3, β4, and β6 conglutins that were cloned into the expression vector, pET28b. [file Image_1.TIF]

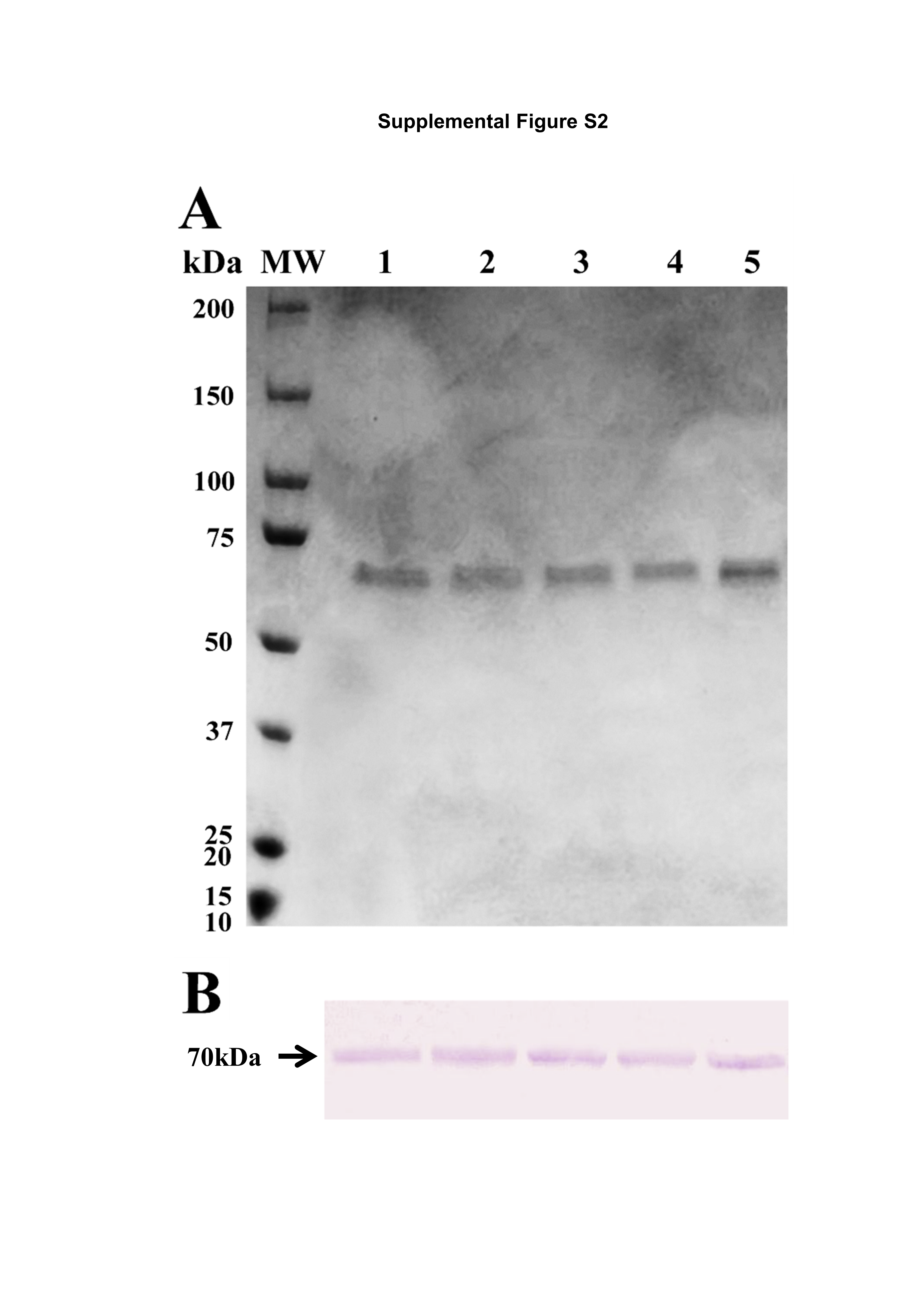

Supplement: FIGURE S2 — Purification and confirmation of recombinant β1- to β4- and β6-conglutins. (A) Purified proteins (10 μg per sample) were separated by SDS–PAGE analyses to indicate a single protein band (6xHis-tag) of approximately 65 kDa at high purity level (>95%). (B) Immunoblot confirmation using anti-β-conglutin protein antibody. The arrow indicates the correct sized band. [file Image_2.TIF]

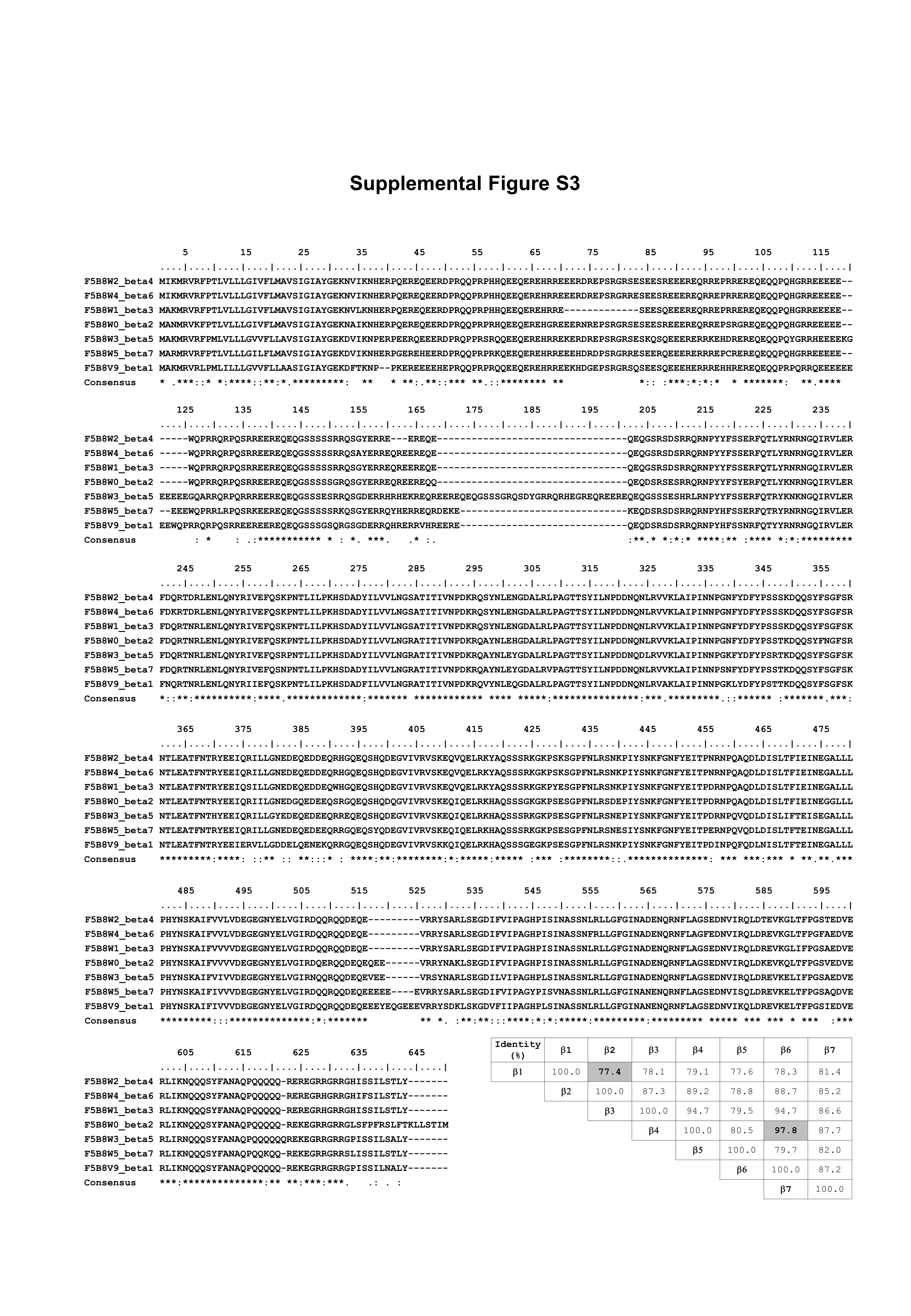

Supplement: FIGURE S3 — Comparison of the NLL β-conglutin protein sequences. Alignment of the seven β-conglutin proteins identified in NLL, and comparison of the level of variability among them. Similarity percentages for each compared-pair of sequences are described in the table below. The lowest and the highest percentages of similarity are highlighted with gray color. [file Image_3.TIF]

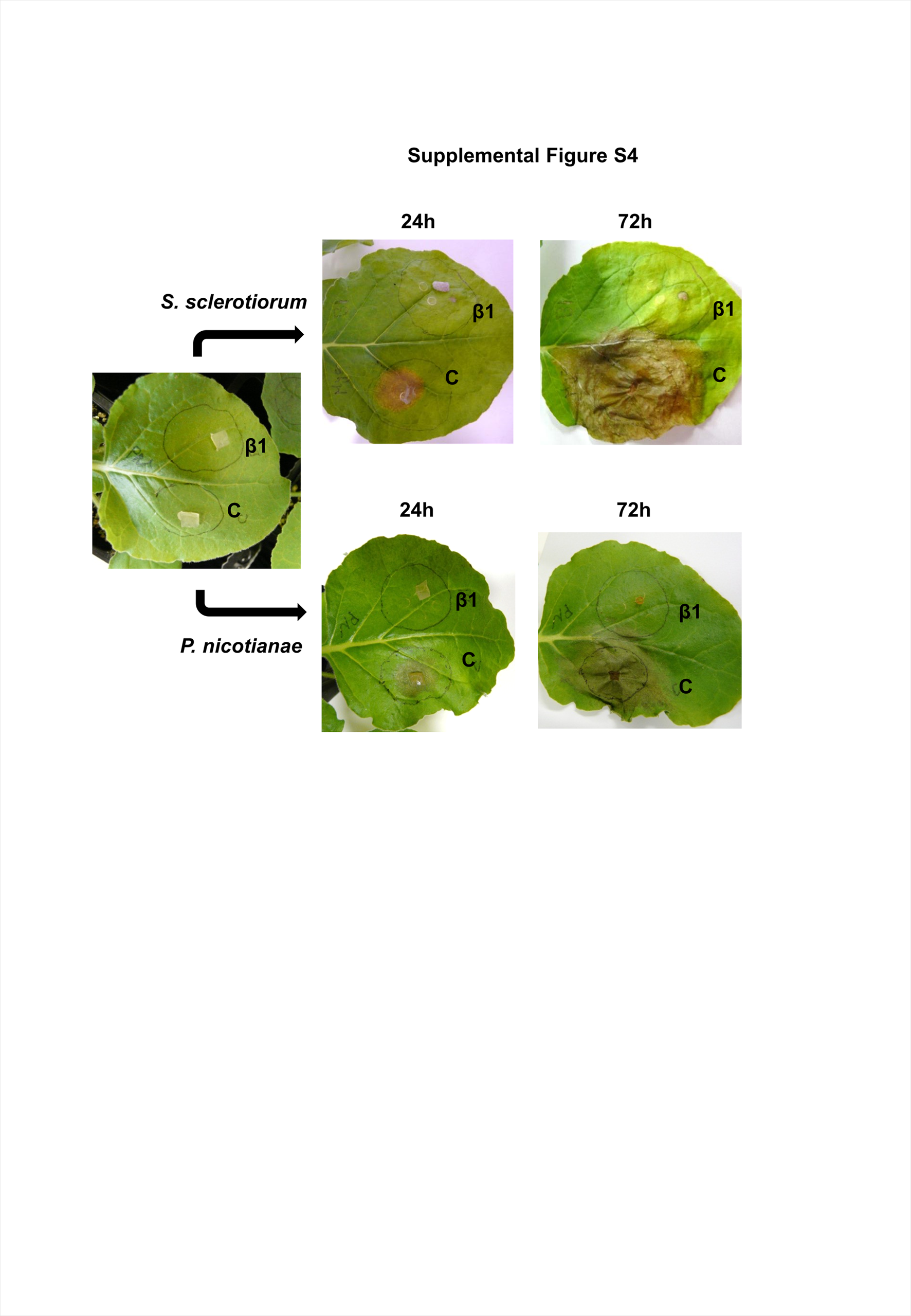

Supplement: FIGURE S4 — Recombinant β1-conglutin exhibits in planta anti-fungal and oomycete activity. Shown are representative images of Agrobacterium infiltrated N. benthamiana leaves expressing recombinant β1-conglutin proteins and subsequently inoculated with either S. sclerotorium or P. nicotianae. The experiment was repeated three times with similar results. [file Image_4.TIF]

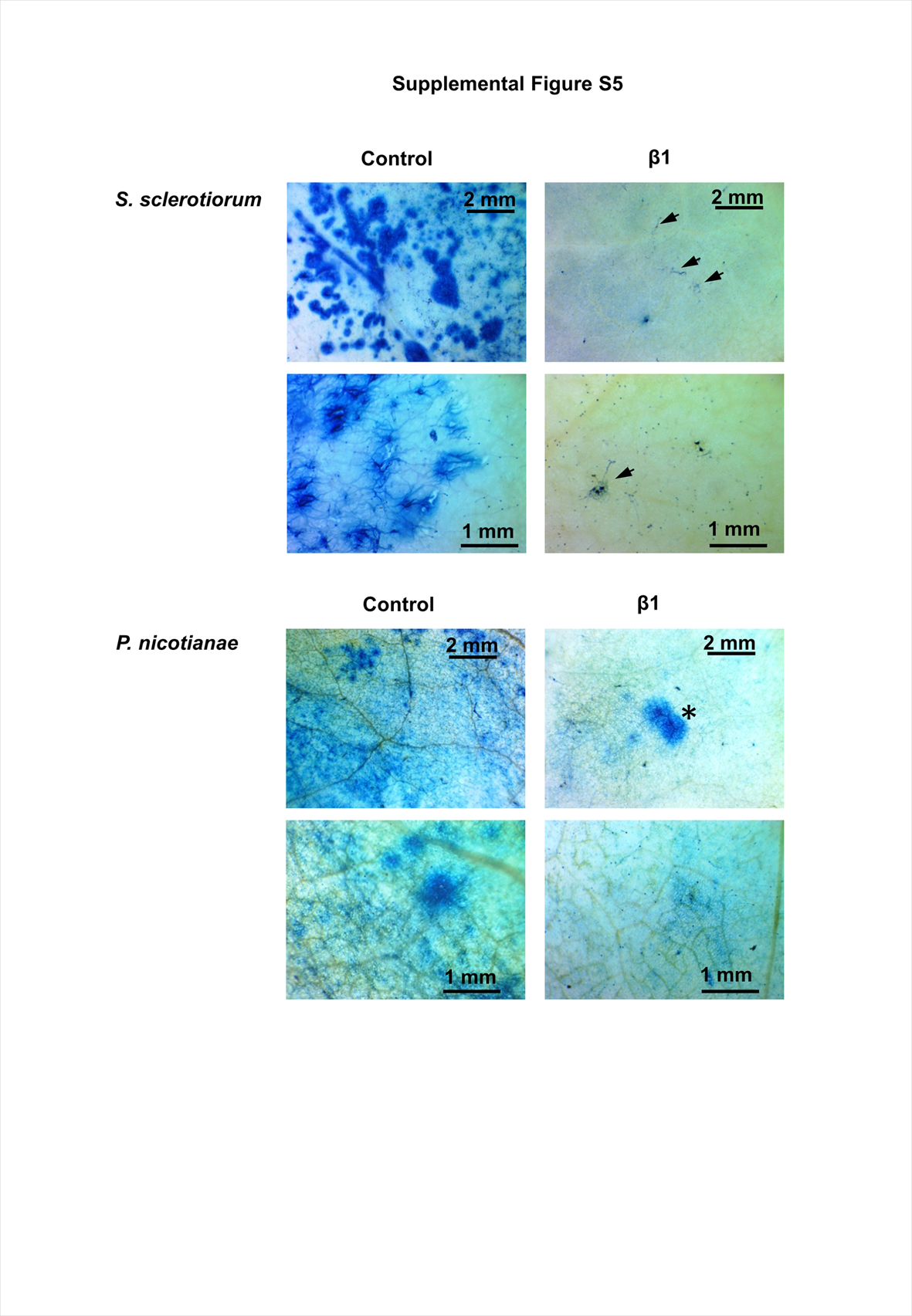

Supplement: FIGURE S5 — Recombinant β1-conglutin reduces pathogen growth and pathogen induced cell death in planta. Shown are representative images of Agrobacterium infiltrated N. benthamiana leaves expressing recombinant β1-conglutin proteins and subsequently inoculated either S. sclerotorium or P. nicotianae. Trypan blue staining was performed to visualize hyphal growth and cell death. Arrows point to hyphae, asterisk marks inoculation site. [file Image_5.TIF]

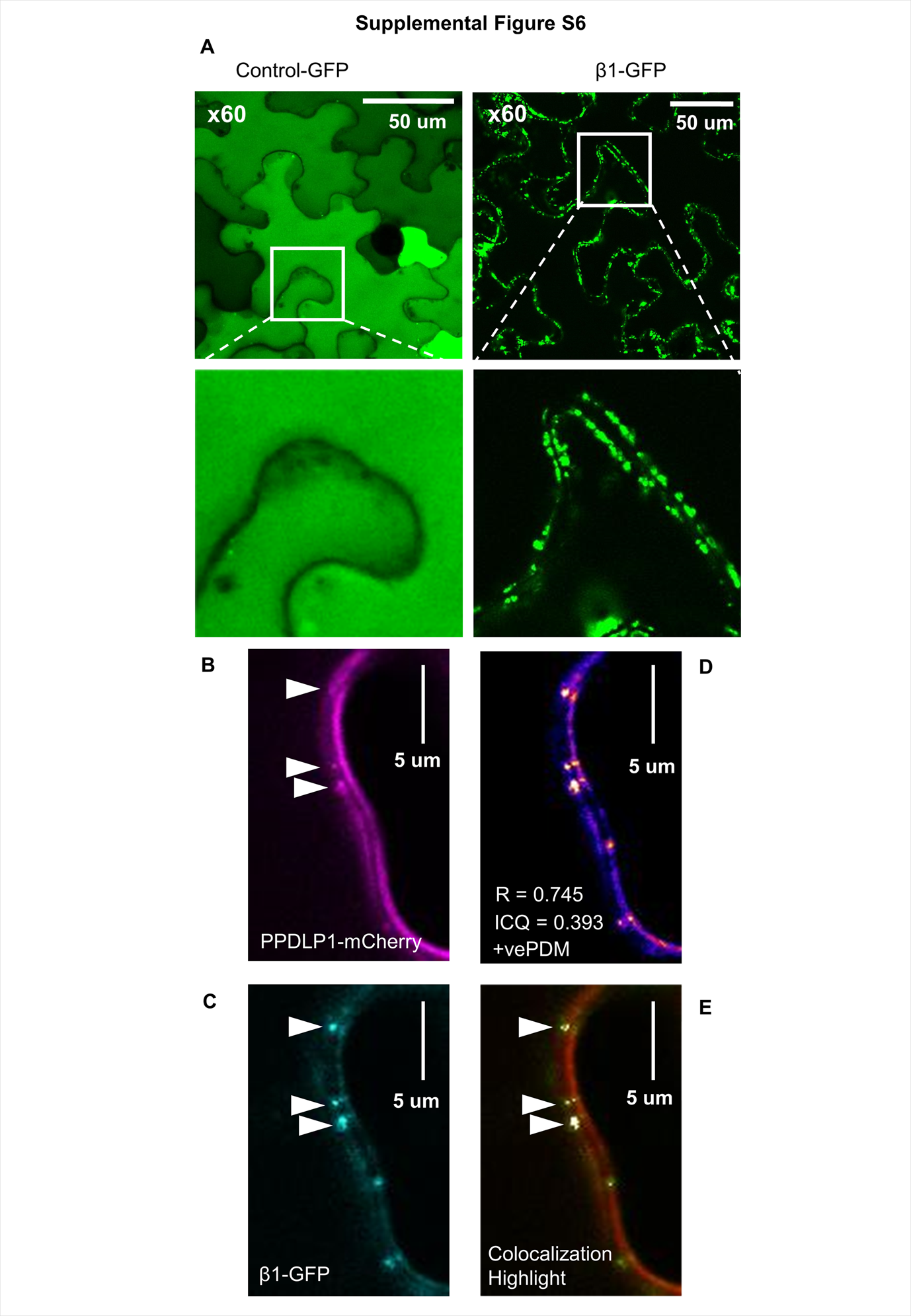

Supplement: FIGURE S6 — β1-conglutin is localized to the cell surface and plasmodesmata. (A) Confocal images of tobacco epidermis cell expressing GFP alone or β1-GFP. Insert: β1-GFP shows punctate labeling at the cell surface. (B–E) Single-slice confocal images of co-expression β1-GFP with the plasmodesmata marker PPDLP1-mCherry after transient expression in N. benthamiana; (B) PDLP1-mCherry, (C) β1-GFP, (D) Image showing pixel pairs that have a positive PDM value equal to the value (intensity of B- mean B intensity) ∗ (intensity of C-mean C intensity) as described in Li et al. (2004), (E) merge of (B,C) with highlighted co-localized pixels. ICQ, Intensity correlation quotient; R = Mandel’s overlap coefficient. 60× immersion objective. [file Image_6.TIF]
